# Hydrogen sulfide–dependent activation of human sulfide quinone oxidoreductase

**DOI:** 10.1016/j.jbc.2025.110681

**Published:** 2025-09-03

**Authors:** Joseph V. Roman, Yashvi Shukla, David A. Hanna, Mackenzie Mackson, Tsz-Leung To, Vamsi Mootha, Ruma Banerjee

**Affiliations:** 1 Department of Biological Chemistry, University of Michigan, Ann Arbor, Michigan, USA; 2 Broad Institute, Cambridge, Massachusetts, USA; 3 Howard Hughes Medical Institute, Boston, Massachusetts, USA; 4 Department of Molecular Biology, Massachusetts General Hospital, Boston, Massachusetts, USA

**Keywords:** sulfide, enzyme, redox, enzyme inactivation, sulfide quinone oxidoreductase

## Abstract

Hydrogen sulfide (H_2_S) is a respiratory poison and also a product of our own metabolism. The toxicity of H_2_S is mitigated by the activity of mitochondrial sulfide quinone oxidoreductase (SQOR), which oxidizes H_2_S while concomitantly reducing coenzyme Q. An unusual cysteine trisulfide cofactor distinguishes SQOR from other members of the flavin disulfide reductase superfamily. In the opening step of the catalytic cycle, an estimated 10^5^-fold rate enhancement is afforded by nucleophilic addition of the sulfide anion to the trisulfide *versus* a disulfide cofactor. The source of the bridging sulfane sulfur in the trisulfide and its mechanism of installation are, however, unknown. We report that H_2_S exposure (100 ppm corresponding to 20 μM dissolved H_2_S, 24 h) increases SQOR activity five- and twofold in human colon adenocarcinoma (HT-29) and transformed endothelial (EA.hy926) cells, respectively. Since activation is not accompanied by a corresponding increase in SQOR protein levels, we conclude that it involves a post-translational mechanism. CRISPR knockdowns of the sulfurtransferases (mercaptopyruvate sulfurtransferase and thiosulfate sulfurtransferase) rule out their involvement in SQOR activation. A combination of pharmacological inhibition and cystine supplementation studies points to the role of H_2_S rather than low molecular weight persulfides in regulating SQOR. We posit that the solvent accessibility and reactivity of the trisulfide make SQOR vulnerable to reversible inhibition. Our study supports a model for trisulfide installation and activation *via* cysteine oxidation and sulfide addition and reveals a heretofore unrecognized mechanism for autoregulating SQOR by H_2_S on demand.

Hydrogen sulfide (H_2_S) is an environmental poison that inhibits complex IV in the electron transport chain (1). H_2_S is also a product of sulfur metabolism and is synthesized by the transsulfuration enzymes cystathionine-β-synthase (CBS) (2) and cystathionine-γ-lyase (CTH) (3) and by mercaptopyruvate sulfurtransferase (MPST) (4). The cytotoxicity of H_2_S is mitigated by its high turnover catalyzed by the mitochondrial inner membrane enzyme, sulfide quinone oxidoreductase (SQOR) (5), which commits H_2_S to thiosulfate and sulfate formation (6, 7). The electrons released during H_2_S oxidation feed into the electron transport chain *via* coenzyme Q (CoQ), a cosubstrate for SQOR. Hence, in addition to being a respiratory toxin, H_2_S is also a respiratory substrate (8), which depending on intracellular conditions, leads to the utilization of O_2_ and/or fumarate as terminal electron acceptors (9, 10).

SQOR is a flavin adenine dinucleotide (FAD)–dependent enzyme that belongs to the flavin disulfide reductase superfamily, whose members harbor a signature cysteine disulfide cofactor in the active site (11). Human SQOR, on the other hand, has an uncommon trisulfide variant of this redox cofactor motif, that is, with an extra sulfane sulfur atom bridging the Cys-201 and Cys-379 thiols (Fig. 1*A*) (12, 13). Computational modeling and molecular dynamics simulations ascribe a 10^5^-fold rate enhancement to the trisulfide over the disulfide, predicting a substantive contribution of this unusual cofactor to the catalytic efficiency of SQOR (14). Recombinant human SQOR expressed in *Escherichia coli* is purified with a full complement of the trisulfide, that is, with a stoichiometry of 1 mol sulfane sulfur per mole SQOR, and does not exhibit inactivation over the time course of the aerobic *in vitro* assay (12). The origin of the bridging sulfur and the mechanism of its installation are, however, unknown.

**Figure 1:**
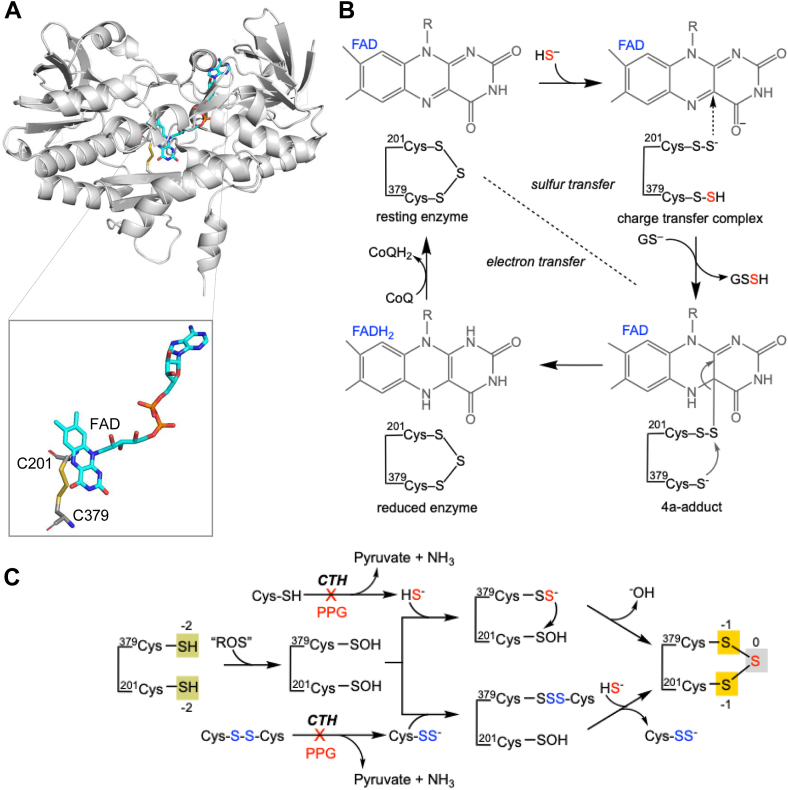
**Structure and catalytic activity of SQOR**. *A,* structure of human SQOR (Protein Data Bank code: 6OI6) showing a close-up of the trisulfide cofactor adjacent to FAD in the active site. *B*, proposed reaction mechanism for human SQOR. *C*, scheme showing possible mechanisms for installation of the bridging sulfane sulfur in the active site trisulfide of SQOR, which involves a net four electron oxidation starting from cysteine as denoted by the numbers above the boxes in the dithiol and trisulfide states. The sulfane sulfur could be derived from H_2_S or a LMW persulfide. In this scheme, CTH is shown as an example of an intracellular source for the sulfane sulfur precursor. PPG denotes propargylglycine, a suicide inhibitor of CTH. CTH, cystathionine-γ-lyase; FAD, flavin adenine dinucleotide; H_2_S, hydrogen sulfide; LMW, low molecular weight; SQOR, sulfide quinone oxidoreductase.

The catalytic cycle of SQOR opens with the nucleophilic attack of the sulfide anion on the trisulfide, forming a charge transfer complex involving a bis-persulfide intermediate (Fig. 1*B*). Of the two cysteine persulfides (Cys-SSHs), ^379^Cys-SSH is solvent exposed, whereas ^201^Cys-SSH is proximal to and engaged in a charge-transfer complex with FAD. To complete the sulfur transfer half reaction, the outer sulfur on ^379^Cys-SSH is transferred to a thiophilic acceptor, primarily GSH in cells (15), whereas a C4a adduct is formed between ^201^Cys-SS^-^ and FAD. Nucleophilic attack by the Cys-379 thiolate on the C4a intermediate reforms the trisulfide and concomitantly reduces FAD. The second half reaction closes with the transfer of electrons from FADH_2_ to CoQ. Due to its promiscuity, SQOR can accommodate a range of sulfane sulfur acceptors besides GSH, including a second mole of sulfide, methanethiol, or sulfite, or the amino acids cysteine or homocysteine (16).

While oligosulfides such as trisulfides are seen in natural products such as diallyltrisulfide in garlic and the malodorous dimethyltrisulfide, a trisulfide post-translational modification is uncommon in nature (17). A protein-bound trisulfide is formed as an intermediate in the microbial sulfate reduction pathway and subsequently undergoes a four-electron reduction to sulfide (18). Trisulfides can be inadvertently introduced in disulfide-containing pharmaceutical proteins during the manufacturing process, which contributes to undesired product heterogeneity. In addition to parameters like cell density and time of harvest, the concentrations of cysteine and H_2_S in the bioreactor medium are correlated with trisulfide formation (19, 20). A noncatalytic trisulfide with no discernible impact on the function or pharmacokinetics of human growth hormone has been reported (21, 22). Similarly, a trisulfide in erythropoietin has little impact on its structure or stability (23). In contrast, the trisulfide in SQOR is essential for catalysis, and dismantling it by cyanolysis renders SQOR inactive, forming a ^379^Cys *N*-(^201^Cys-disulfanyl)-methanimido thiolate species whose crystal structure has been described (14).

Due to its reactivity and solvent exposure, the trisulfide itself is also susceptible to nucleophilic attack by other small (cyanide, methanethiol, and sulfite) and large (GSH) nucleophiles besides sulfide, which inhibit SQOR reversibly (14, 16). Restoration of the resting trisulfide can occur either *via* dissociation of the nucleophile (GSH and sulfite), that is, reversal of the addition reaction, or *via* resolution of the inhibited enzyme by sulfide (cyanide and methanethiol). The solvent exposure and reactivity of the trisulfide raises the question as to whether its reversible dismantling represents a regulatory strategy for attenuating SQOR activity and increasing it on demand, that is, in response to increasing sulfide. Alternatively, its location in the mitochondrial inner membrane could render SQOR susceptible to reversible oxidative inhibition, which in turn could be repaired when sulfide levels increase, reforming the trisulfide.

Formation of a trisulfide involves a net four-electron oxidation starting from cysteines. Each of the cysteine thiols (Cys-201 and Cys-379) changes from the −2 to −1 oxidation state while the bridging sulfane sulfur is zero valent, and could, in principle, be installed in reactions involving a low molecular weight (LMW) persulfide and/or sulfide (Fig. 1*C*). Cys-SSH is formed by the α−β elimination of cystine catalyzed by the transsulfuration pathway enzymes (24, 25), whereas GSSH is a product of the SQOR reaction (7). A plausible mechanism for trisulfide installation involves oxidation of Cys-379 and Cys-201 followed by nucleophilic addition by a persulfide and/or sulfide (Fig. 1*C*). In contrast, the involvement of a cysteine disulfide as a starting point for trisulfide assembly is unlikely because of the ∼4.6 Å distance between the sulfur atoms on Cys-379 and Cys-201 in human SQOR (12). Furthermore, the bimolecular rate constant for the addition of sulfide to sulfenic acid (1 × 10^5^ M^−1^s^−1^) is vastly favored over its addition to cysteine disulfide (0.6 M^−1^s^−1^) (26, 27).

An alternative to an LMW persulfide is that a sulfurtransferase-bound persulfide serves as the sulfane sulfur donor. Human sulfurtransferases that could potentially serve as sulfur donors to SQOR include MPST (4), thiosulfate sulfurtransferase (TST) (28) and thiosulfate sulfurtransferase–like domain–containing (TSTD1) protein (29). Of these, MPST and TST are mitochondrial, whereas TSTD1 is cytoplasmic. MPST catalyzes sulfur transfer from 3-mercaptopyruvate, generating pyruvate and an active site cysteine persulfide (4). The enzyme-bound sulfane sulfur is stable and has been characterized by crystallography; it is subsequently transferred to thiophilic acceptors, such as GSH, cysteine, and thioredoxin, forming the corresponding persulfide (30). Despite the persulfide being sequestered, MPST reportedly functions as a sulfur donor to a host of other proteins *via* a transpersulfidation reaction (31). TST is a component of the mitochondrial sulfide oxidation pathway and catalyzes the interconversion of sulfite and thiosulfate using GSSH and GSH, respectively (7). TST reportedly also harbors protein transpersulfidation activity (32), donating a sulfane sulfur atom to very long chain acetyl-CoA dehydrogenase (33). TSTD1 is the least studied of the three sulfurtransferases and can transfer sulfane sulfur from thiosulfate to thioredoxin, albeit inefficiently (29). Unlike MPST and TST, the active site of TSTD1 is solvent exposed, and its role in trisulfide installation is unlikely since it would have to occur in the cytoplasm, that is, prior to the mitochondrial import of SQOR.

In this study, we demonstrate that chronic H_2_S exposure (100 ppm H_2_S corresponding to 20 μM dissolved sulfide) in a custom sulfide growth chamber (34) increases SQOR activity in human colorectal adenocarcinoma (HT-29) and transformed human endothelial (EA.hy926) cells ∼5-fold and 2-fold, respectively. Surprisingly, following removal of sulfide from the atmosphere, SQOR activity returns to basal levels. Since the H_2_S-induced increase in activity is not correlated with an increase in SQOR levels, the reversible activation is attributed to an increase in the proportion of the trisulfide-containing enzyme. Using a combination of CRISPR knockdowns (KDs) of candidate sulfurtransferases, cystine supplementation, and pharmacological inhibitors, we conclude that H_2_S is primarily responsible for activating SQOR. Our study reveals a heretofore unknown cellular capacity for holding SQOR in an inert state and activating it on demand.

## Results

### Chronic H2S exposure increases SQOR activity

**Figure 2:**
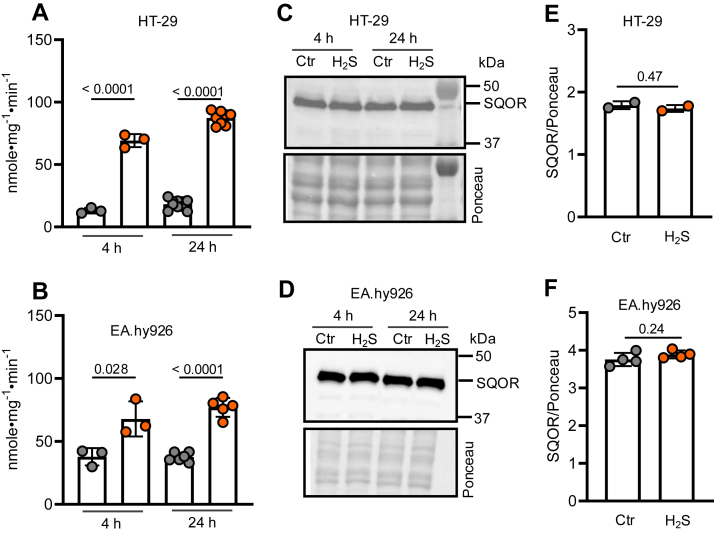
**H_2_S exposure increases SQOR activity in cells**. *A*–*B*, SQOR activity in HT-29 (*A*) and EA.hy926 (*B*) cells grown in the presence (*orange*) or absence (*gray*) of 100 ppm H_2_S for 4 or 24 h. *C*–*E*, Western blot (*C* and *D*) and quantitation (*E* and *F*) of SQOR expression in HT-29 cells (*C* and *E*) and EA.hy926 cells (*D* and *F*) exposed to 100 ppm H_2_S. Data are represented as mean ± SD with each data point representing an independent experiment and the mean of at least two technical replicates. Significance was determined using the two-tailed *t* test. H_2_S, hydrogen sulfide; SQOR, sulfide quinone oxidoreductase.

**Figure 3:**
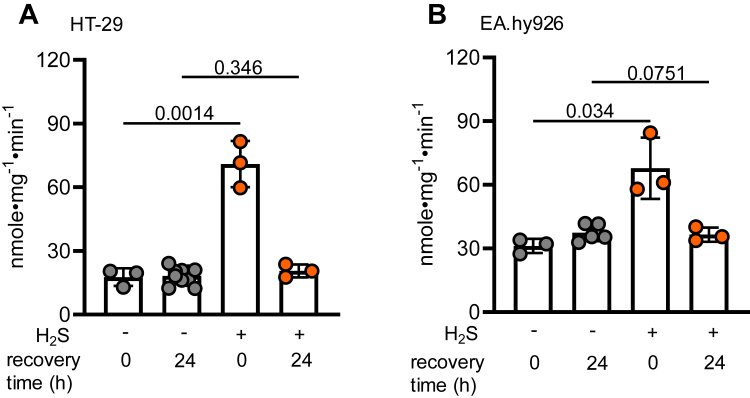
**Return to basal SQOR activity following sulfide-induced activation**. *A*–*B*, SQOR activity in HT-29 (*A*) and EA.hy926 (*B*) cells exposed to 100 ppm H_2_S for 6 h followed by 24 h of recovery. Each data point represents an independent experiment with at least two technical replicates. Data are represented as mean ± SD. Significance was determined using the two-tailed *t* test. H_2_S, hydrogen sulfide; SQOR, sulfide quinone oxidoreductase.

SQOR activity was measured in HT-29 and EA.hy926 cells grown for up to 24 h in a sulfide incubator in the presence of 100 ppm H_2_S (34). SQOR activity increased fivefold in HT-29 cells from 13.0 ± 2.2 to 69.2 ± 5.2 nmole•mg^−1^•min^−1^ after 4 h and from 18.2 ± 3.8 to 87.3 ± 5.3 nmole•mg^−1^•min^−1^ after 24 h (Fig. 2*A* and Table 1). Similarly, SQOR activity increased ∼2-fold in EA.hy926 cells from 37.8 ± 6.9 to 67.8 ± 13.9 nmole•mg^-1^•min^-1^ in 4 h and from 37.5 ± 3.6 to 77.0 ± 7.5 nmole•mg^−1^•min^−1^ over 24 h (Fig. 2*B* and Table 1). Interestingly, the increase in activity was not accompanied by a change in SQOR protein levels in either cell line (Fig. 2, *C*–*F*). Sulfide-induced activation of SQOR was reversible and returned to basal levels within 24 h after cells were moved to a regular growth chamber from a sulfide chamber (Fig. 3). Since a change in protein level was not associated with the observed increase in activity, we posit that a significant proportion of SQOR exists in an inactive state in which the trisulfide cofactor is dismantled and can be rebuilt in response to H_2_S exposure. Since the magnitude of H_2_S-induced SQOR activation is larger in HT-29 cells, further studies were largely focused on this cell line.

**Table 1:** SQOR activity in sulfide-grown HT-29 and EA.hy926 cells

Culture conditions	SQOR-specific activity in HT29 cells (nmole•mg^-1^•min^-1^)	SQOR-specific activity in EA.hy926 cells (nmole•mg^-1^•min^-1^)
4 h air	13.0 ± 2.2 (n = 3)	37.8 ± 6.8 (n = 3)
100 ppm H_2_S, 4 h	69.2 ± 5.2 (n = 3)	67.8 ± 13.9 (n = 3)
6 h air	17.7 ± 4.1 (n = 3)	32.3 ± 3.2 (n = 3)
100 ppm H_2_S, 6 h	70.9 ± 10.9 (n = 3)	67.8 ± 14.5 (n = 3)
24 h air	18.2 ± 3.8 (n = 10)	35.5 ± 3.6 (n = 5)
100 ppm H_2_S, 24 h	87.3 ± 5.3 (n = 8)	77.0 ± 7.5 (n = 5)

### Effects of cyanide and sulfite on SQOR in cells

**Figure 4:**
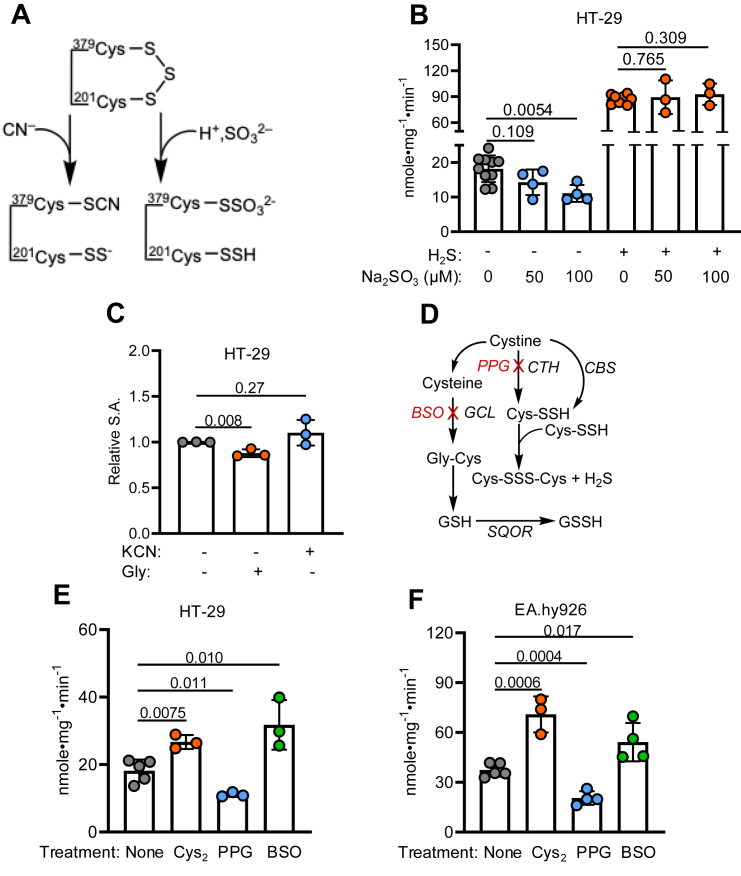
**Effect of various treatments on SQOR activity**. *A*, postulated mechanism of *in vitro* inhibition of SQOR by cyanide or sulfite. *B*, SQOR activity in HT-29 cells grown for 24 h in the presence of 50 or 100 μM sodium sulfite in the presence (*orange*) or absence (*blue*) of 100 ppm H_2_S. *C*, relative SQOR-specific activity (SA) in HT-29 cells treated with glycine (10 mM, *orange*) or potassium cyanide (500 μM, *blue)* for 24 h relative to untreated control cells (*gray*). *D*, scheme showing targets of PPG and BSO in relation to Cys-SSH synthesis. *E*–*F*, SQOR activity in HT-29 cells (*E*) or EA.hy926 cells (*F*) treated with 500 μM cystine (Cys_2_), 2.5 mM PPG, or 20 μM BSO compared with untreated cells. Each data point is the average of at least two technical replicates and represents an independent experiment. Data are presented as mean ± SD. Significance was determined using a two-tailed *t* test. BSO, buthionine sulfoximine; Cys-SSH, cysteine persulfide; H_2_S, hydrogen sulfide; PPG, propargylglycine; SQOR, sulfide quinone oxidoreductase.

Both cyanide and sulfite can reversibly dismantle the SQOR trisulfide *in vitro* (Fig. 4*A*) (14, 16). Cyanide has recently been described as a metabolic product of lysosomal glycine oxidation (35), whereas sulfite is an intermediate in the cysteine and sulfide oxidation pathways (6, 36). In the presence of sulfite in normal air, SQOR activity decreased from 18.2 ± 3.8 (control) to 14.3 ± 3.7 (50 μM sulfite) and 11.1 ± 2.4 (100 μM sulfite) nmole•mg^−1^•min^−1^, respectively, consistent with inhibition observed previously under *in vitro* conditions (Fig. 4*B* and Table 2). However, sulfite failed to elicit an effect when cells were grown in the presence of H_2_S (100 ppm). The 40,000-fold difference in the bimolecular rate constant for the addition of sulfite (1.03 × 10^2^ M^−1^s^−1^) *versus* sulfide (4.0 × 10^6^ M^−1^s^−1^) to the SQOR trisulfide (16) likely accounts for the predominance of the sulfide effect over sulfite under our experimental conditions. When glycine (10 mM) was added to the culture medium to stimulate cyanide synthesis (35), a small (1.1-fold) but statistically significant inhibitory effect on SQOR activity was seen (Fig. 4*C*). In contrast, potassium cyanide (500 μM) had no effect on SQOR activity (Fig. 4*C*) but led to acidification as revealed by yellowing of the Phenol Red pH indicator in the culture medium, signaling increased aerobic glycolysis.

**Table 2:** Effect of cyanide and sulfite on SQOR activity in HT-29 cells

Culture conditions	Specific activity (nmole•mg^-1^•min^-1^)
24 h air^a^	18.2 ± 3.8 (n = 10)
5 μM cystine, 24 h air	29.1 ± 1.1 (n = 3)
50 μM SO_3_^2-^, 24 h air	14.3 ± 3.7 (n = 4)
100 μM SO_3_^2-^, 24 h air	11.1 ± 2.4 (n = 4)
100 ppm H_2_S, 24 h^a^	87.3 ± 5.3 (n = 8)
5 μM cystine, 100 ppm H_2_S, 24 h	124.5 ± 9.8 (n = 3)
50 μM SO_3_^2-^, 100 ppm H_2_S, 24 h	89.4 ± 19.5 (n = 3)
100 μM SO_3_^2-^, 100 ppm H_2_S, 24 h	92.7 ± 9.9 (n = 3)

a These values are from Table 1.

### Sulfurtransferases do not activate SQOR

**Figure 5:**
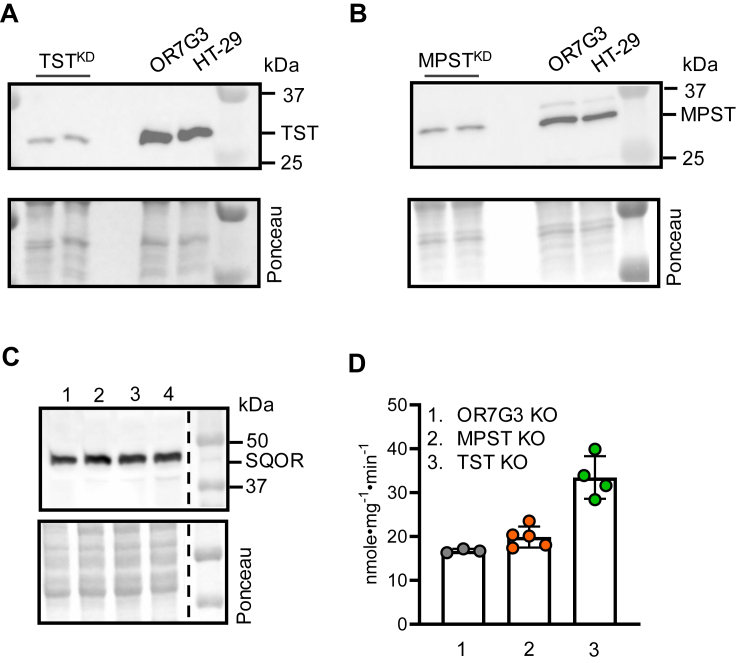
**Effect of sulfurtransferase knockdowns on SQOR activity**. *A*–*B*, Western blot analysis of MPST (*A*) and TST (*B*) in control (HT-29 and OR7G3) *versus* the corresponding CRISPR knockdown cell lines. Ponceau staining is shown as equal loading controls. *C*, representative Western blot of SQOR in control HT-29 cells (*lane* 1) compared with HT-29 cells with CRISPR knockdown of OR7G3 (*lane* 2), MPST (*lane* 3), and TST (*lane* 4). The image was spliced at the position indicated by the *dashed line* to remove a lane that is not relevant to this study. *D*, SQOR activity in HT-29 cells with the indicated CRISPR knockdowns. Each data point represents an independent experiment and is the mean ± SD of at least two technical replicates. Significance was determined using a two-tailed *t* test. MPST, mercaptopyruvate sulfurtransferase; OR7G3, olfactory receptor 7G3; SQOR, sulfide quinone oxidoreductase; TST, thiosulfate sulfurtransferase.

We next evaluated the individual sulfurtransferases as potential sulfane sulfur sources. CRISPR KDs of the two mitochondrial sulfurtransferases as well as of OR7G3 (olfactory receptor 7G3, which is not expressed in HT-29 cells and serves as a cutting control) were generated in HT-29 cells (Fig. 5, *A* and *B*). The KDs did not affect SQOR expression (Fig. 5*C*). Basal SQOR activity was also unaffected in the OR7G3 (16.7 ± 0.5 nmole•mg^−1^•min^−1^) and MPST (19.9 ± 2.3 nmole•mg^−1^•min^−1^) KD lines, but surprisingly, was ∼2-fold higher in the TST KD (33.5 ± 4.9 nmole•mg^−1^•min^−1^) (Fig. 5*D* and Table 3). TST catalyzes reversible sulfur transfer between thiosulfate and GSSH (28), which is a product of the SQOR reaction (Fig. 1*B*) (15). Thus, TST KD could increase the GSSH pool, which can activate SQOR either directly or more likely, *via* elimination of H_2_S (2GSSH → GSSSG + H_2_S). Collectively, these data rule out a direct involvement of the sulfurtransferases tested here in SQOR activation.

**Table 3:** SQOR activity in HT-29 and EA.hy926 cell lysates

Cell line	Specific activity (nmole•mg^-1^•min^-1^) control	Specific activity (nmole•mg^-1^•min^-1^) + cystine	Specific activity (nmole•mg^-1^•min^-1^) + PPG	Specific activity (nmole•mg^-1^•min^-1^) + BSO
HT-29	18.2 ± 3.3 (n = 5)	26.7 ± 2.1 (n = 3)	11.1 ± 0.6 (n = 3)	31.7 ± 7.9 (n = 3)
HT-29^OR7G3^	16.7 ± 0.5 (n = 3)	29.5 ± 2.2 (n = 3)	10.9 ± 1.2 (n = 3)	28.9 ± 2.8 (n = 3)
HT-29^MPST KD^	19.9 ± 2.3 (n = 5)	29.2 ± 1.9 (n = 3)	15.1 ± 1.1 (n = 3)	34.2 ± 1.3 (n = 3)
HT-29^TST KD^	33.5 ± 4.9 (n = 4)	51.6 ± 5.9 (n = 3)	17.0 ± 4.3 (n = 4)	51.2 ± 6.9 (n = 3)
EA.hy 926	36.4 ± 3.7 (n = 4)	70.9 ± 10.8 (n = 3)	20.5 ± 4.1 (n = 4)	54.2 ± 11.5 (n = 4)

### Cystine increases SQOR activity

**Figure 6:**
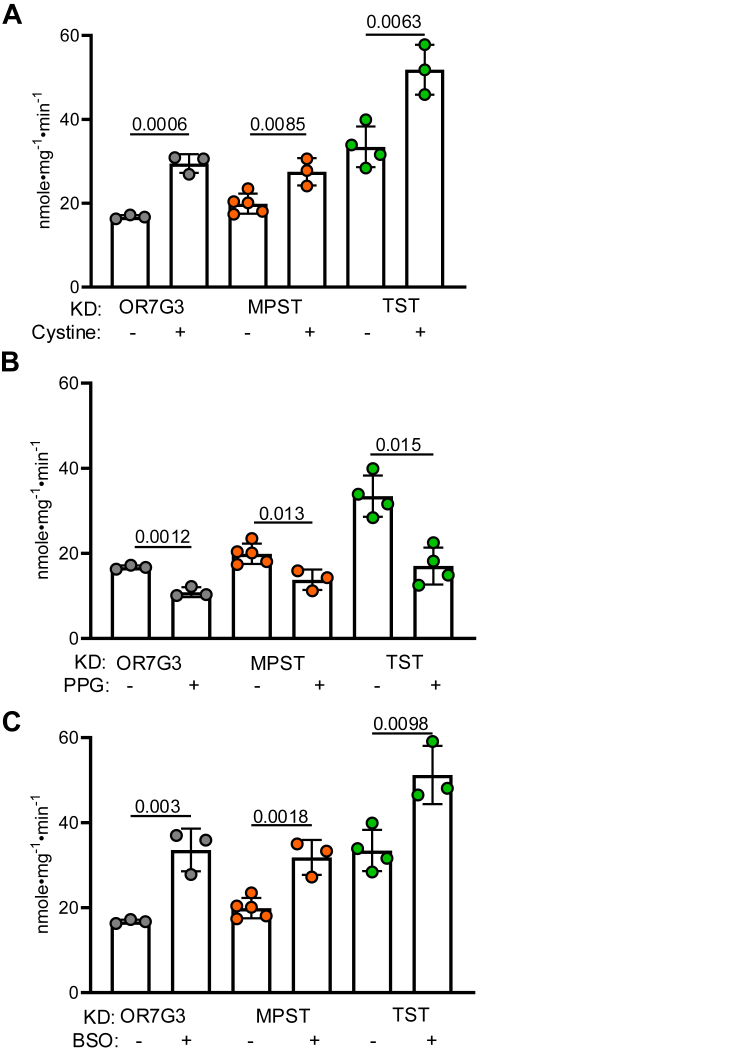
**Effect of cystine, PPG, and BSO on SQOR activity in HT-29 cells with MPST and TST knockdowns**. *A*, SQOR activity in cells grown in the presence of cystine (750 μM final concentration) for 24 h. *B*–*C*, SQOR activity in cells treated for 96 h with 2.5 mM PPG (*B*) or 20 μM BSO (*C*). Each data point represents an independent experiment and is the mean ± SD of at least two technical replicates. Significance was determined using the two-tailed *t* test. BSO, buthionine sulfoximine; MPST, mercaptopyruvate sulfurtransferase; PPG, ropargylglycine; SQOR, sulfide quinone oxidoreductase; TST, thiosulfate sulfurtransferase.

Cystine can be converted to Cys-SSH by CBS or CTH (Fig. 4*D*), and extracellular cystine supplementation increases intracellular LMW persulfides (24, 25). Since Cys-SSH is metastable, it is also a source of H_2_S (Fig. 4*D*). Cystine can also stimulate H_2_S biogenesis directly, that is, following intracellular reduction to cysteine (37), in an α−β elimination reaction catalyzed by CBS (2) or CTH (3). Supplementation of the culture medium with cystine (resulting in an increase from 250 to 750 μM) stimulated SQOR activity 1.5-fold in HT-29 and ∼2-fold in EA.hy926 cells (Fig. 4, *E* and *F* and Table 3). Cystine supplementation also increased SQOR activity in HT-29^MPST KD^ and HT-29^TST KD^ cells (Fig. 6*A* and Table 3).

The role of CTH-dependent cystine metabolism in activating SQOR was assessed with propargylglycine (PPG), a mechanism-based inhibitor (38). PPG was used at a concentration of 2.5 mM for 96 h, a concentration reported to inhibit CTH by ∼90% (39) decreased SQOR activity 1.6-fold in HT-29 and 1.8-fold in EA.hy926 cells (Fig. 4, *E* and *F* and Table 3). PPG similarly decreased SQOR activity in HT-29^MPST KD^ and HT-29^TST KD^ cells (Fig. 6*B* and Table 3). It is important to note that while CBS is expressed in EA.hy926, it is not detectable in HT-29 cells (40).

### Inhibition of GSH synthesis increases SQOR activity

Since GSH synthesis is typically limited by cysteine levels, cystine supplementation could increase GSH, and, thereby, GSSH levels (Fig. 4*D*). To evaluate whether GSH synthesis influences SQOR activation in response to cystine supplementation, cells were treated with buthionine sulfoximine (BSO, 20 μM, 96 h), an inhibitor of γ-glutamyl cysteine ligase (41). BSO treatment (20 μM, 96 h) increased SQOR activity 1.7-fold in HT-29 cells and 1.5-fold in EA.hy926 cells (Fig. 4, *E* and *F* and Table 3). BSO similarly increased SQOR activity in HT-29^MPST KD^ and HT-29^TST KD^ cells (Fig. 6*C*). Since TST utilizes GSSH as a substrate, our data rule out a direct role for GSSH in activating SQOR based on the comparable effects of BSO in control *versus* HT-29^TST KD^ cells. The data so far implicate an LMW persulfide other than GSSH and/or H_2_S in the cellular activation of SQOR, which were distinguished next.

### Increase in LMW persulfides in low cysteine-treated HT-29 cells

**Figure 7:**
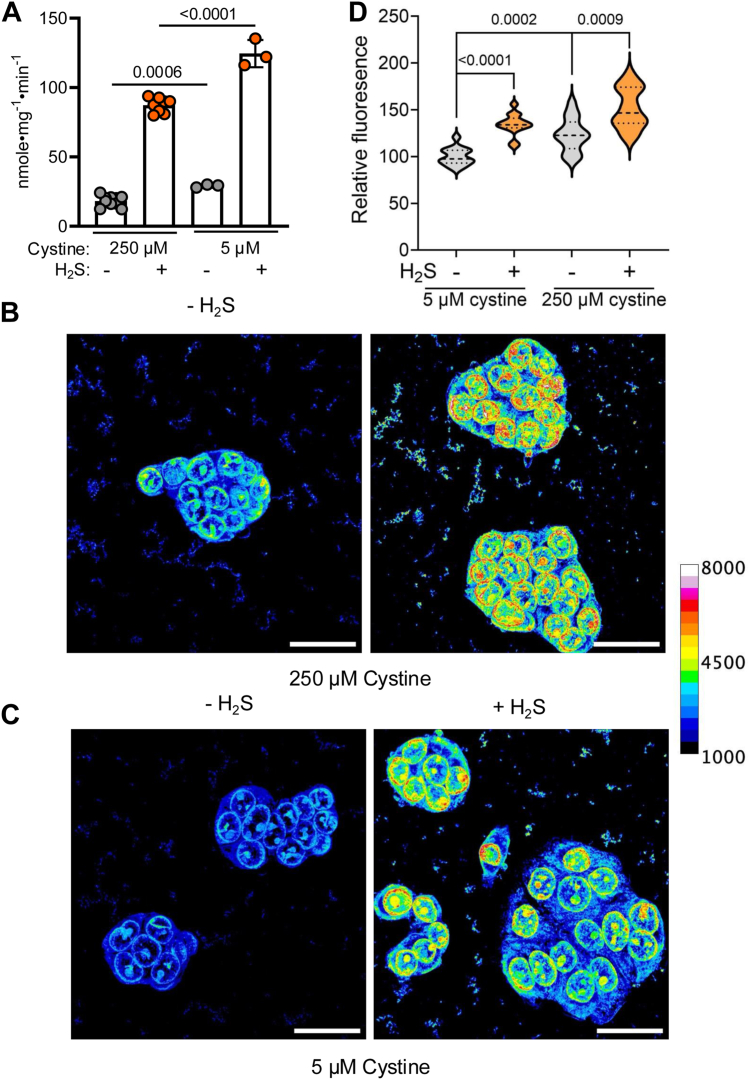
**Effect of low cystine on SQOR activity and LMW persulfides**. *A*, SQOR activity in HT-29 cells grown in the presence (*orange*) or absence (*gray*) of 100 ppm H_2_S and with low (5 μM) or standard (250 μM) cystine in the medium. *B*–*C*, representative images showing the difference in SSP4 fluorescence intensity in HT-29 cells grown with standard (*B*) or low (*C*) cystine in the culture medium and ± H_2_S exposure. The calibration bar shows that the pixel intensity is within the detector’s range, that is, 0 to 65,535, and uses a false color scale to visualize changes in SSP4 fluorescence. The scale bar represents 30 μm. *D*, violin plots comparing the mean fluorescence for cells from 14 different images from three independent experiments grown in the presence (*orange*) and absence (*gray*) of H_2_S and low or standard cystine. Significance was determined using an unpaired two-tailed *t* test. H_2_S, hydrogen sulfide; LMW, low molecular weight; SQOR, sulfide quinone oxidoreductase.

We compared the effect of low (5 μM) *versus* 250 μM cystine present in RPMI medium on SQOR activity and intracellular LMW persulfide levels. Surprisingly, H_2_S exposure (100 ppm, 24 h) induced a similar fourfold increase in SQOR activity in cells grown in the presence of low *versus* standard cystine medium (Fig. 7*A*). Thus, SQOR activity increased from 18.2 ± 3.6 to 87.3 ± 5.3 nmole•mg^−1^•min^−1^ in standard RPMI medium and from 29.1 ± 1.1 to 124.5 ± 9.8 nmole•mg^−1^•min^−1^ in medium containing 5 μM cystine. Notably, cells grown in the low cystine medium exhibited a statistically significant 1.5-fold increase in SQOR activity both in the absence and presence of H_2_S, indicating that this condition was protective for basal SQOR activity.

SSP4 is a fluorophore, which, based on leaving group p*K*a values, is predicted to react preferentially with LMW *versus* protein-bound sulfane sulfur species to liberate fluorescein (25, 42). HT-29 cells grown in standard RPMI medium exhibited a modest increase in fluorescence compared with cells grown in medium containing 5 μM cystine (Fig. 7, *B*–*D*). H_2_S (100 ppm, 24 h) induced only modest increases in SSP4 fluorescence under both cell culture conditions (Fig. 7*D*). These data strongly point to a direct role for sulfide rather than Cys-SSH in activating SQOR.

## Discussion

**Figure 8:**
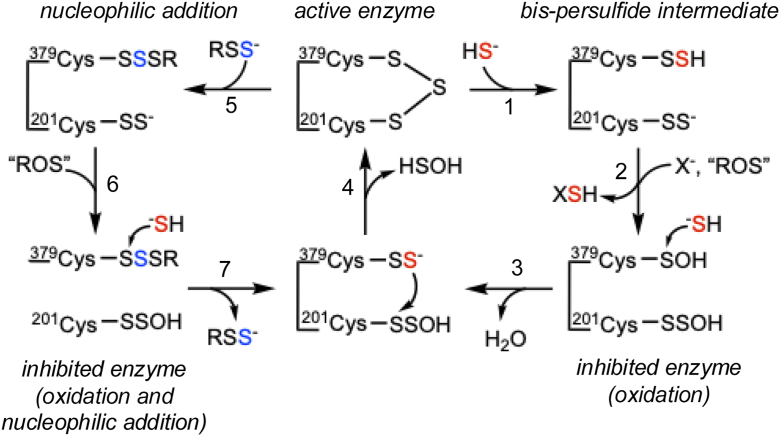
**Scheme showing mechanism of inactivation/reactivation of SQOR**. The active trisulfide enzyme is converted to the bis-persulfide intermediate (1), which following transfer of the sulfane sulfur to an acceptor (X^-^) can undergo oxidation (2) to form inactive oxidized SQOR. In the presence of H_2_S, the sulfenic acid at Cys-379 undergoes nucleophilic addition, leading to elimination of a water and formation of a persulfide (3), from which the active trisulfide form can be rebuilt (4). Alternatively, the active form of SQOR can undergo nucleophilic addition, shown here with an LMW persulfide (5) followed by oxidation (6) to give inactive enzyme. In the presence of H_2_S, the persulfide is eliminated from Cys-379 (7), leading to the reformation of the trisulfide (4). The oxidant is depicted generically as ROS and could be H_2_O_2_. LMW, low molecular weight; ROS, reactive oxygen species; SQOR, sulfide quinone oxidoreductase.

The intrinsic reactivity of a solvent-accessible and electrophilic trisulfide cofactor renders SQOR vulnerable to nucleophilic and/or oxidative inactivation (Fig. 8) and raises the question as to whether reversible inhibition represents a heretofore unrecognized strategy for activating SQOR on demand. Based on Western blot analysis, SQOR levels are estimated to be ∼40% higher in EA.hy926 compared with HT-29 cells (43). The two- *versus* fivefold activation elicited by H_2_S reveals that 50% of SQOR in EA.hy926 cells but only 20% in HT-29 cells is active under basal cell culture conditions (Fig. 2, *A* and *B*). The gain in SQOR activity is not accompanied by a corresponding increase in protein levels (Fig. 2, *C* and *D*) and occurs within 4 h, that is, the earliest time point at which the concentrations of sulfide in the culture medium and the atmosphere are equilibrated under our experimental conditions (34). The activation is transient, and cells revert to basal SQOR activity levels upon cessation of sulfide exposure (Fig. 3). Our data are consistent with the model that a sizeable proportion of SQOR is inactive but primed to regenerate the active enzyme in the presence of H_2_S.

Although the source of sulfane sulfur for trisulfide installation and activation is unknown, we speculate they might be the same. The sulfur in cysteine thiol and H_2_S is in the −2 oxidation state, precluding direct interaction between them. Therefore, the sulfur donor and/or acceptor must first be oxidized in order to build the trisulfide (Fig. 1*C*). We propose that active SQOR is susceptible to inadvertent oxidation during turnover, for example, after sulfur transfer from the bis-persulfide intermediate (Fig. 8, *steps* 1 and 2). Although Cys-379 is more exposed than Cys-201, which is adjacent to the isoalloxazine ring of the FAD cofactor (Fig. 1*A*), we posit that oxidation at both cysteines occurs in the reversibly inhibited enzyme. Mechanistically, the proposed oxidation at both cysteines is analogous to that for reversible inhibition of SQOR by 2 mol of cyanide per mole of trisulfide (14). The problem that arises with oxidation of a single cysteine, for example, Cys-379 to cysteine sulfenic acid is that H_2_S-independent reformation of the trisulfide could occur by nucleophilic addition of the Cys-201 persulfide and elimination of water. As an alternative, reversible inhibition could be initiated by spurious addition of a nucleophile, for example, GSH or an LMW persulfide, leading to a mixed disulfide at Cys-379, followed by oxidation at Cys-201, leading to a persulfenic acid (Fig. 8, *steps* 5 and 6). In the presence of sulfide, the trisulfide could then be recovered (Fig. 8, *step* 4).

The model helps explain several of our observations. Cystine supplementation increased SQOR activity modestly (Fig. 4*E*), whereas MPST KD, which would decrease enzyme-bound Cys-SSH, had no effect (Fig. 5*D*) in HT-29 cells. Based on detailed kinetic analyses, cystine supplementation is predicted to impact H_2_S synthesis from cysteine (following reduction of cystine) more substantively than Cys-SSH synthesis from cystine (25). This prediction is borne out by the rather modest increase in LMW persulfides as detected by SSP4 fluorescence in cells despite a 50-fold (5 μM *versus* 250 μM) increase in extracellular cystine (Fig. 7*D*). The *k*_cat_/*K*_*M*_ for CTH is 21-fold and 4-fold higher than for CBS for Cys-SSH and H_2_S synthesis, respectively (2, 3, 25), making it primarily responsible for their syntheses (Fig. 1*C*). In HT-29 cells, which express CTH but not CBS, both Cys-SSH and H_2_S synthesis should be inhibited by PPG. The magnitude of inhibition by PPG was similar in HT-29 and EA.hy926 cells, consistent with the dominant contribution of CTH to modulating SQOR activity (Fig. 4, *E* and *F*). Collectively, the kinetic parameters strongly point to H_2_S rather than Cys-SSH as the likely metabolite responsible for SQOR activation, as discussed later.

The increase in SQOR activity by BSO (Fig. 4, *E* and *F*), which lowers intracellular GSH, while paradoxical, is consistent with our model. We predict that by decreasing cysteine consumption for GSH synthesis, BSO increases the available cysteine pool (Fig. 4*D*), which in turn stimulates H_2_S synthesis by CTH. Furthermore, by decreasing the concentration of GSH, a cosubstrate for SQOR, BSO could promote oxidation at both cysteines in the bis-persulfide intermediate (a mechanistic alternative that is not included in Fig. 8 for the sake of simplicity). It is less likely that the spurious addition of GSH to the trisulfide is attenuated by BSO, given the modest bimolecular rate constant for this reaction (*k*_on_ = 0.38 M^−1^ s^−1^) (16).

The increase in SQOR activity in the TST^KD^ cells is surprising and could result from decreased utilization of GSSH, leading to increased synthesis of the trisulfide, GSSSG and H_2_S, with the latter activating SQOR. GSSH (and Cys-SSH) are metastable because of the dual reactivity of persulfides as electrophiles and nucleophiles. The *k*_cat_/*K*_*M*_ for GSSH for TST (8.6 × 10^5^ M^−1^s^−1^ (28)) is sixfold higher than for ETHE1 (1.4 × 10^5^ M^−1^s^−1^ (44)), an enzyme in the sulfide oxidation pathway that also uses GSSH as a substrate. Furthermore, since Cys-SSH is an alternate substrate for TST (45), decreased consumption in TST KD cells could increase H_2_S elimination. In *Cupriavidus pinatubonensis* JMP134, the SQOR and TST homologs are fused in a single polypeptide. Interestingly, SQOR activity is increased in the fusion protein when the rhodanese domain is inactivated (46).

A direct role for H_2_S rather than an LMW persulfide is supported by (i) the large increase in SQOR activity in cells grown in an H_2_S atmosphere (Fig. 2, *A* and *B*), (ii) the modest increase in intracellular LMW persulfides under these conditions (Fig. 7*D*), and (iii) the significantly higher basal SQOR activity in cells grown in the presence of 5 *versus* 250 μM cystine (Fig. 7*A*).

In summary, we report that the intrinsic reactivity of the SQOR trisulfide cofactor represents a catalytic vulnerability as well as a regulatory strategy for responding to demand, that is, an increase in H_2_S. The regulation is clearly exerted at a post-translational level, and we posit that it involves elaboration of the active trisulfide cofactor from an oxidized, inactive holding state. Our study implicates exogenous as well as CTH-derived H_2_S in SQOR activation, and we propose that a similar mechanism, that is, sulfide addition to oxidized cysteine sulfenic acid could be involved in the initial installation of the trisulfide. It is unclear whether the regulatory strategy reflects a stochastic inactivation of SQOR in the cellular milieu because of the solvent exposure of the electrophilic trisulfide cofactor or if a specific mechanism exists to convert active to the inactive enzyme when sulfide flux is low. A limitation of our study is that we were unable to directly trace the source of the sulfane sulfur or complement our cellular studies with *in vitro* characterization of trisulfide formation because of the instability of SQOR in the dithiol state. If inactivation of SQOR at low concentrations of H_2_S represents a regulatory strategy, its rationale is presently unclear. Alternatively, inactivation could merely be a consequence of the enhanced electrophilicity of the trisulfide *versus* a disulfide cofactor. In either case, reactivation *via* reformation of the trisulfide cofactor represents a unique mechanism for regulating sulfide oxidation.

## Experimental procedures

### Materials

RPMI1640, Dulbecco's modified Eagle's medium, penicillin/streptomycin solution (10,000 U/ml penicillin, 10,000 μg/ml streptomycin), fetal bovine serum (FBS), and puromycin sodium salt were all purchased from Gibco. Phenol red and serum-free RPMI medium (11835030), bovine serum albumin, BioLite 60 mm cell culture–treated dishes (130181), RPMI medium lacking methionine, cysteine, and glutamine were purchased from Thermo Fisher Scientific. Potassium phosphate, monobasic, sodium sulfite, cystine, propargyl glycine (P7888), cetyltrimethylammonium bromide, Tris-base, disodium sulfide nonahydrate (103386788), and protease inhibitor cocktail (P8340) were purchased from MilliporeSigma. BSO (14484) and CoQ_1_ (18741) were purchased from Cayman Chemicals. Polyvinylidene difluoride membrane (1620177), 10% acrylamide gels, Clarity Western peroxide reagent, and Clarity Western luminol/enhancer reagent (1705061) were purchased from Bio-Rad. Glass bottom dishes (35 mm; 81158) and 8-well μ-slide dishes (80826) were purchased from Ibidi. 1,2-Dicaproyl-*sn*-glycero-3-phosphocholine was purchased from Avanti Polar Lipids (850305). The SQOR (17256-1-AP; Proteintech), TST (66018-1-Ig; Proteintech Group), and MPST (Novus Biologicals; NBP154734) antibodies were purchased from the indicated vendors. The antirabbit-horseradish peroxidase antibody was from Abcam (Ab6721). EDTA was purchased from Acros Organics. Nonident P-40 substitute was purchased from Fluka (74385). Bel-Art Stir and Add Cuvette Mixer were purchased from Bel-Art (F37752-0001). HT-29 cells and EA.hy926 (immortalized human vascular endothelial) cells were purchased from the American Type Culture Collection. pLentiCRISPRv2 (Addgene #52961), pMD2.G (Addgene #12259), and psPAX2 (Addgene #12260) were from Addgene. HT-29 and EA.hy926 cells were from the American Type Culture Collection and were routinely tested in the laboratory and established to be mycoplasma free.

### Cell culture

HT-29 cells were grown in 6 cm plates at 37 °C in an atmosphere of 21% O_2_ and 5% CO_2_. Cells were seeded at ∼1.5 million cells per plate in RPMI1640 medium with 2 mM glutamine and 10% FBS and 1000 U/ml penicillin and 1 mg/ml streptomycin. The medium was changed every 48 h, and cells were harvested on the fifth day when confluency reached ∼90%. EA.hy926 cells were grown under similar conditions except in high-glucose Dulbecco's modified Eagle's medium supplemented with 10% FBS and 1000 U/ml penicillin and 1 mg/ml streptomycin. Cells were harvested after aspirating the medium, washing with 2 ml cold 1× PBS, and scraping in 1 ml PBS. The cell suspension was transferred to preweighed 1.7 ml Eppendorf tubes, centrifuged at 1600*g* for 5 min, following which the supernatant was discarded. The wet weight of cells was determined, and pellets were either frozen at −80 °C or used immediately for SQOR assays.

Alternatively, cells were seeded at ∼3.0 million per 6 cm plate and cultured for 24 h without H_2_S and then grown for up to 24 h in a sulfide growth chamber in an atmosphere containing 100 ppm H_2_S and a humidified mixture of 95% breathing air and 5% CO_2_ as described (34). For the recovery experiments, two plates of cells were harvested after 6 h to establish the level of SQOR activation, whereas a second set of two plates was moved to a standard cell culture incubator (21% O_2_, 5% CO_2_) after a medium change and allowed to recover for 24 h prior to harvest.

When used, PPG (2.5 mM) or BSO (20 μM) was added to the culture medium, and cells were grown with fresh aliquots of the inhibitor added every day for a total of 4 days. The duration of the treatment was chosen to accommodate SQOR turnover and new synthesis in the presence of the inhibitors. The effect of high cystine on SQOR activity was evaluated by adding 27 μl cystine (from a 75 mM stock solution prepared in 150 mM NaOH) to 4 ml of culture medium to obtain a final concentration of 750 μM cystine (RPMI medium contains 250 μM cystine) 24 h prior to harvest. Control plates received 27 μl of 150 mM NaOH. The effect of low cystine was evaluated by growing cells for 24 h in methionine-, cysteine-, and glutamine-free RPMI1640 medium, supplemented with 10% FBS and 1000 U/ml penicillin and 1 mg/ml streptomycin. The medium also contained 100 μM methionine, 2 mM glutamine, and either 5 or 250 μM cystine was added. The following day, the medium was changed, and cells were moved into the sulfide chamber (100 ppm H_2_S) and grown for an additional 24 h prior to harvesting.

### SQOR assay in cell lysates

Cell pellets were resuspended in lysis buffer (4 μl per mg of wet weight). The lysis buffer contained 50 mM Tris (pH 7.4), 200 mM NaCl, 1 mM EDTA, 1 mM MgCl_2_, 1% ^v^/_v_ Nonident P-40 substitute, and 10 μl/ml protease inhibitor cocktail. Cells were lysed by vortexing for 1 min followed by a 30 s incubation on ice. This process was then repeated two more times prior to freezing in dry ice for 10 min. Frozen cells were thawed in a room temperature water bath for 3 min, and the vortex/freezing process was repeated once. The resulting lysate was centrifuged at 10,000*g* for 10 min, and the supernatant was carefully transferred to a fresh 1.7 ml Eppendorf tube. The protein content was determined by the Bradford assay, using bovine serum albumin as a standard. SQOR activity was measured as described previously (7). Briefly, in a 1 ml cuvette, 100 mM potassium phosphate (pH 7.4), containing 70 μM CoQ_1_, 800 μM sodium sulfite, 0.03% 1,2-dicaproyl-*sn*-glycero-3-phosphocholine, and 0.06 μg/ml BSA was incubated for 3 min. Then, 50 μg of total protein (typically in 5–10 μl) was added using a Bel-Art Stir and Add Cuvette Mixer. The decrease in absorbance at 278 nm was monitored for 60 s, which represented the background rate of CoQ reduction by all enzymes present in the cell lysate. SQOR-dependent CoQ reduction was initiated by the addition of 150 μM Na_2_S. The specific activity of SQOR was estimated using a Δε_278 nm_ of 12,000 M^-1^•cm^-1^. The data for the background (*i*.*e*., minus Na_2_S) reactions across all the experiments were averaged and compared with SQOR activity in response to individual treatments for both HT-29 and EA.hy926 cells. Similarly, the SQOR-specific activity in cells exposed to 100 ppm H_2_S (0 and 24 h) was averaged and compared with the treatment condition, for example, sulfite.

### Western blot analysis

HT-29 and EA.hy926 cells were lysed, and total protein concentration was determined as described previously. Total protein from HT-29 (30 μg) or EA.hy926 (20 μg) cells was mixed with sample loading buffer containing 62.5 mM Tris (pH 7.5), 1.5% SDS (w/v), 8% glycerol (v/v), 150 mM DTT, and 0.03% bromophenol blue (w/v), and incubated at 95 °C for 5 min. Samples were then centrifuged for 2 min at 10,000*g* prior to loading onto a 10% polyacrylamide gel. The gel was run at 100 V for 10 min followed by an additional 45 min at 125 V. Proteins were then transferred to a polyvinylidene difluoride membrane using a Bio-Rad transblot turbo apparatus set to 100 V for 30 min. Protein-bound membranes were then blocked in 1× Tris-buffered saline with Tween-20 (TBST) (20 mM Tris [pH 7.5], 150 mM NaCl, and 0.3% Tween-20 [v/v]) containing 5% milk for 1 h. Blocked membranes were then transferred to a heat-sealed pouch containing 3 ml TBST + milk with 1:1000-fold dilution of anti-SQRDL, 1:2000 dilution of anti-MPST, or 1:1000 dilution of anti-TST antibody and incubated overnight at 4 °C. The following day, membranes were washed three times for 5 min with 1× TBST prior to incubating for 1 h at room temperature with 10 ml of TBST + 5% milk containing a 1:10,000-fold dilution of anti-rabbit (MPST and SQOR) or a 1:10,000 dilution of anti-mouse antibodies tethered to horseradish peroxidase. Membranes were then washed three more times for 5 min with 1× TBST followed by three washes with TBS (20 mM Tris [pH 7.5], 150 mM NaCl). Membranes were incubated for 2 min in a 50:50 mixture (1.2 ml) of Clarity Western peroxide reagent:Clarity Western luminol/enhancer reagent immediately prior to visualizing chemiluminescence *via* a Bio-Rad ChemiDoc MP Imaging System.

### CRISPR KD of sulfurtransferases

The following single guide RNAs for each gene of interest were ordered as complementary oligonucleotides from Integrated DNA Technologies and cloned into pLentiCRISPRv2 according to the vendor’s (Addgene) protocol.

OR7G3 (Forward) 5ʹ-CACCGGGTGAAACAGATGTCGACCA-3ʹ

OR7G3 (Reverse): 5ʹ-AAACTGGTCGACATCTGTTTCACCC-3ʹ

MPST (Forward): 5ʹ-CACCGGGCGTCGTAGATCACGACGT-3ʹ

MPST (Reverse): 5ʹ-AAACACGTCGTGATCTACGACGCCC-3ʹ

TST (Forward): 5ʹ-CACCGATGTCAAAGAAAGAGGCGCC-3ʹ

TST (Reverse): 5ʹ-AAACGGCGCCTCTTTCTTTGACATC-3ʹ

Lentivirus was harvested from human embryonic kidney 293T cells according to the vendor’s (Addgene) protocol, using the pMD2.G and psPAX2 envelope and packaging plasmids. HT-29 cells were seeded in 6-well plates at ∼600,000 cells per well and grown to a confluency of 70% prior to transfection using 1 ml of medium from the human embryonic kidney 293T cells containing the pLentiCRISPrv2 construct with the guide sequences for the sulfurtransferase KDs. HT-29 cells were then grown for 2 days prior to changing the medium and introducing 1 μg/ml puromycin to select for cells containing the pLentiCRISPRv2.

### Persulfide imaging

HT29 cells were seeded at 10,000 cells per well in 8-well Ibidi μ-slide dishes or at 25,000 cells per well in Ibidi 35-mm glass bottom dishes and cultured overnight in standard RPMI1640 with 2 mM glutamine supplemented with 10% FBS and 1000 U/ml penicillin and 1 mg/ml streptomycin. After 24 h, the medium was replaced with RPMI containing normal (250 μM) or low (5 μM) cystine, before moving the samples into the sulfide chamber (±100 ppm H_2_S) for 24 h. Then, the medium was replaced with Phenol red, and serum-free RPMI medium supplemented with 0.5 mM cetyltrimethylammonium bromide and cells were stained with 10 μM SSP4 for 15 to 30 min and imaged for H_2_S- and cystine-dependent changes in fluorescein fluorescence.

Z-stack images were captured using the Zeiss LSM 980 Airyscan 2 microscope with CO_2_ and temperature regulation, a 488 nm laser, a 63× oil objective with a 1.4 numerical aperture, and a 5-megapixel Axiocam 705 monochrome CMOS camera and Airyscan detector for super-resolution imaging. The images were captured using the Airyscan MPLX imaging mode, acquiring 70 nm pixels, and achieving ∼140 nm resolution. All images were analyzed using FIJI. For this, maximum intensity projections of the fluorescence intensity images were made, and equal intensity thresholding was used for all analyzed images to segment the cells. The segmented cells had their mean pixel intensity quantified, generating a mean pixel intensity for each image, which was used to generate violin plots in Prism.

## Data availability

All data are contained within the article and the supporting information section.

## Conflict of interest

R. B. is a consultant for Zyphore Therapeutics and Alnylum Pharmaceuticals. All the other authors declare that they have no conflicts of interest with the contents of this article.
